# Pulmonary Toxicity and Recovery from Inhalation of Manual Metal Arc Stainless Steel Welding Fumes in Rats

**DOI:** 10.5487/TR.2008.24.2.119

**Published:** 2008-06-01

**Authors:** Mi-Jin Yang, Jin-Sung Kim, Young-Su Yang, Jae-Woo Cho, Seong-Bong Choi, Yong-Hyun Chung, Yong-Bum Kim, Kyu-Hyuk Cho, Chae-Woong Lim, Choong-Yong Kim, Chang-Woo Song

**Affiliations:** 15 Division of Inhalation Toxicology, Korea Institute of Toxicology, Korea Research Institute of Chemical Technology, P.O BOX 123, Yuseong, Daejeon, 305-343 Korea; 25 Occupational Safety & Health Research Institute, Daejeon, 305-343 Korea; 35 Department of Pathology, College of Veterinary Medicine, Chonbuk National University, Jeonju, 561-756 Korea

**Keywords:** Welding fume inhalation, Manual metal arc stainless steel, Bronchoalveolar lavage, Rats

## Abstract

The objectives of this study were to examine the lung injury and inflammation caused by manual metal arc stainless steel (MMA-SS) welding fume inhalation and to evaluate the recovery process. Sprague-Dawley rats were exposed to MMA-SS welding fumes for 2 h per day in a whole-body exposure chamber, with a total suspended particulate (TSP) concentration of 51.4 ± 2.8 mg/m^3^ (low dose) or 84.6 ± 2.9 mg/m^3^ (high dose) for 30 days. The animals were sacrificed after 30 days of exposure as well as after a 30-day recovery period. To assess the inflammatory or injury responses, cellular and biochemical parameters as well as cytokines were assayed in the bronchoalveolar lavage fluid (BALF). MMA-SS welding fume exposure led to a significant elevation in the number of alveolar macrophages (AM) and polymorphonuclear cells (PMN). Additionary, the values of β-nacetyl glucosaminidase (β-NAG) and lactate dehydrogenase (LDH) in the BALF were increased in the exposed group when compared to controls. After 30 days of recovery from exposure, a significant reduction in inflammatory parameters of BALF was observed between the exposed and recovered groups. Slight, but significant elevations were noted in the number of AM and PMN in the recovered groups, and AM that had been ingested fume particles still remain in the lungs. In conclusion, these results indicated that welding fumes induced inflammatory responses and cytotoxicity in the lungs of exposed rats. Fume particles were not fully cleared from lungs even after a 30-day recovery period.

## INTRODUCTION

More than 5 million workers worldwide, employed as full-time welders, are exposed to welding aerosols repeatedly on a daily basis (Antonini *et al*., 2007). The majority of welders are employed in shipbuilding, transport equipment manufacturing, building construction, petrochemical, mining, and metallurgical industries. These workers are exposed to fumes and gases that may be hazardous to their health. Numerous epidemiological studies have been revealed the adverse health effects associated with welding, which conclude airway irritation, metal fume fever, chemical pneumonitis, chronic bronchitis, decrements in pulmonary function, and pneumoconiosis (Niosh, 1988; Sferlazza *et al*., 1991). The marked radiographic abnormalities of the welder's pneumoconiosis display large opacities that gradually disappeared due to the absence of fume exposure (Sohn *et al*., 1994).

Arc welding is the principal industrial process which bound metals using electricity that passed between the work pieces and an electrode (Howden *et al*., 1988). The arc produces a temperature of about 3600°C at the electrode tip and melts part of the metal and electrode. This produces a pool of molten metal and in turn welding fumes were generated via vaporization of the core metal and flux components of the electrode. Welding fumes are a complex gaseous and aerosol by-product of metals, metal oxides, and other chemical species, such as iron (Fe), chromium (Cr), manganese (Mn), and nickel (Ni).

Numerous studies have been performed to evaluate the toxicity of welding fumes. MMA-SS fumes were more cytotoxic to macrophages and induced a greater release of reactive oxygen species than fumes from gas metal arc-mild steel (GMA-MS), and consequently they caused more pneumotoxic effects in rats (Antonini *et al.*, 1996, 1997). Previously, three phases of lung fibrosis were established in rats induced by welding fume exposure (Yu *et al.*, 2001). Early delicate fibrosis was observed following welding fume exposure at high concentrations from 15 days. This progressed into the perivascular and peribronchiolar regions by 30 days, and corresponded to an elevation in inflammatory cells and markers. Fibrosis was preventable when the exposure was stopped at 30 days and allowed to recover, as demonstrated in a previous study (Yu *et al.*, 2003b). These experimental data indicated that the critical point for the induction and recovery of lung fibrosis was 30 days.

Therefore, the goals of the current study were to assess the degree of lung inflammation and injury by welding fume inhalation, to investigate the recovery process from pulmonary damage induced by welding fumes, and to elucidate the progress of fibrosis and its recovery in lungs 30 days after exposure was stopped. To evaluate the pulmonary responses caused by the welding fume inhalation and the recovery process, a variety of cellular and biochemical parameters, such as albumin, LDH, β-NAG, TNF-α and IL-1β, were measured in BALF and histopathological examinations were also performed.

## MATERIALDS AND METHODS

***Generation and analyses of MMA-SS welding fumes.*** Welding fumes were generated as described previously (Yu *et al.*, 2007). The welding fume generation system, which used in this study was divided into three different sections: an automated robotic welding fume generator, two animal exposure chambers with different fume concentrations, and a fume collector. In the fume generator, a robotic arm clamped a consumable welding rod (KST 308, 2.6 × 300 mm, Korea Welding Electrode Co. Ltd, Seoul, Republic of Korea) and approached the stainless steel base plate (SUS 304, 2.5 cm thick). When the welding rod and the base plate came into contact, an electric arc was generated, and the produced welding fumes were passed to the exposure chambers (Whole-body type, each 1.5 $m^3$, Dusturbo, Seoul, Republic of Korea). The whole process lasts for 2 min 50 sec. The rat was accommodated in the chambers with purified air supplied through HEPA filters.

Personal sampler (MSA, PA, USA) was used for sampling the welding fumes at a flow rate of 2 liter/min through the exposure chamber. The fume particles were collected from the center of the chamber and captured on mixed cellulose ester (MCE) membrane filters (pore size 0.8 μm, 37 mm diameter, Millipore, MA, USA). The metal composition of the collected fumes was analyzed using an inductively coupled plasma analyzer (Thermojeralash, IRIS, TX, USA) according to Niosh 7300 method (1999). The concentration in the chamber was measured by collecting fumes onto polyvinyl chloride (PVC) membrane filters (pore size 5 μm, 37 mm diameter, PALL GLA-5000, pall cor.) during exposure to welding fume for 2 h.

The gaseous fraction of welding fumes, ozone, nitrous oxide, and nitrous fumes were measured using colorimetric Dräger tubes (Cat No. 6733181, CH 30001, CH 29401, respectively) and sampled by stroking a gas detector pump (6400000, Dräger, Lübeck, Germany) according to the manufacturer's directions. The distribution of particle size within the welding fumes was determined using an Anderson 8-stage cascade impactor (AN-200, SIBATA, Tokyo, Japan) at a flow rate of 28.3 liter/min for 10 min. Chamber concentration, temperature, and humidity were recorded and maintained throughout welding fume exposure.

***Study of MMA-SS welding fume inhalation exposure and recovery.*** Specific pathogen-free (SPF) Sprague-Dawley rats (six-week-old males) were purchased from Charles River Laboratory (Atsuki, Japan), with an average weigh of 164 ± 6 g. They were acclimated for 3 weeks and randomly divided into two group based on the experiment periods as the exposure of 30 days and the recovery of 30 day following exposure. Each group consisted of 6 unexposed, 7 low-dose exposed, and 7 rats exposed to high-dose. The rats were fed Lab diet 5053, watered filtered tap water, and were housed in a HEPA-filtered clean air, viral- and antigen-free room. All the animal facilities in this study were accredited by the Association for Assessment and Accreditation of Laboratory Animal Care International (AAALAC). The rats were exposed to welding fumes at a concentration of 51.4 ± 2.8 $mg/m^3$ (low dose) and 84.6 ± 2.9 $mg/m^3$ (high dose) for 2 h per day without providing food or water, after which they were taken out of the chamber.

***Bronchoalveolar lavage (BAL).*** BAL was carried out on rats from each group as described by Antonini *et*

*al*. (2004). The rats were deeply euthanized with an overdose of *isoflurane* and exsanguinated by severing the abdominal aorta. The left lung was clamped off and the right lungs were first washed out with twice 3 ml aliquots of warm, calcium- and magnesium-free phosphate buffer solution (PBS) (pH 7.4). The two bronchoalveolar lavage fluid (BALF) were centrifuged (500 ×g, 10 min, 4°C) and the resultant cell-free supernatant was analyzed for various biochemical parameters and cytokine levels as described below. The right lungs were further lavaged 8 more times with 3 ml aliquots of PBS and these samples were centrifuged (500 g, 10 min, 4°C) and the supernatant discarded. The cell pellets from all recovered lavages for each rat were combined, resuspended in 1 ml of PBS, and evaluated for cellular parameters.

***Cellular evaluation of BAL cells.*** The cells recovered by BAL were counted and identified. The total BAL cell numbers were measured using a Coulter counter multisizer 3 (Berkman coulter, USA). BAL cells were spun at 800 rpm for 5 min and pelleted onto a slide using a cytospin 4 centrifuge (Thermo-Shandon, Pittsburgh, PA). Cells (300/rat) were differentially counted as alveolar macrophages (AM), polymorphonuclear cells (PMN), and lymphocyte after staining with Wright-giemsa Sure stain.

***Analyses of biochemical parameters and inflammatory cytokines.*** Several parameters were measured as indicators of pulmonary damage within the first acellular supernatant fraction of BALF. (1) Albumin content was assayed to quantitate the increased permeability of the bronchoalveolar capillary barrier. (2) The activity of the lysosomal enzyme, β-n-acetyl glucosaminidase (β-NAG), was measured to determine the release of enzymes from activated or lysed phagocytes. (3) The activity of the cytosolic enzyme, lactate dehydrogenase (LDH), was tested to examine the general cytotoxicity. (4) Alkaline phosphatase (ALP) was analyzed to detect Type 2 alveolar epithelial cell secretory activity. LDH and ALP measurements were performed with DRY-CHEM 3500S (Fuji Film, Tokyo, Japan) and the albumin and β-NAG was determined using TBA 200FR (Toshiba Corporation, Tokyo, Japan), respectively.

The levels of cytokines, tumor necrosis factor-α (TNF-α), and interleukin-1β (IL-1β) were assayed in the first BALF via enzyme linked immunosorbent assays (ELISA). Cytokines concentrations were measured using ELISA kits from a rat TNF-α ELISA kit (Bender MedSystems, CA, USA) and a rat IL-1β ELISA kit (R&D system Inc., MN, USA). The cytokines to be assayed were selected based on their potential role in lung inflammatory and immune responses after welding fume exposure. The measurements of cytokines were performed, according to the procedure provided by themanufactures. Samples and standards were measured in duplicate.

***Organ Weight and Histology.*** After collection of BALF on days 30 and 60, the left lung was excised and weighed, and the right lungs were perfused intratracheally with 10% neutral phosphate buffered formalin (NPBF) and fixed. The heart, liver, kidney, spleen, testis, brain, and nasal turbinate were excised, weighed, and fixed in 10% NPBF for more than 3 days. Nasal pathways were gently flushed via the nasopharyngeal duct with 10% NPBF and fixed for 7 days, decalcified with 10% formic acid for more than 7 days, and stored in NPBF pending processing. Three histological sections were made from the proximal nasal pathway to the distal nasal pathway: incisor teeth, incisive papilla, and molar teeth regions. Specimens were dehydrated and embedded in paraffin, sectioned in 5 ìm slices onto microscope slides, and stained with hematoxylin and eosin (H&E).

***Statistical analysis.*** All results are expressed as the means ± standard deviation of measurement (S.D.). Statistical analyses were carried out with the Path/Tox System (Version 4.2.2, Xybion Medical Systems Corporation, USA). For all parameters, Bartlett's test was performed whether a significant interaction was present or not, Dunn's Rank Sum and ANOVA tests were used to compare control to experimental groups. The significance between each of the individual groups at each time point was analyzed using the t-test. Statistical significance was established when $p < 0.05$, $p < 0.01$.

## RESULTS

***Characterizations of MMA-SS welding fumes.*** The size distributions of the welding fume particles are shown in Table 1. More than 90% of fume particles had

**Table 1.** Size distribution of MMA-SS welding fume particles

| Size (μm) | % sampled | Cumulative (%) |
|---|---|---|
| > 11.0 | 0.35 | 100.00 |
| 11.0~7.0 | 0.74 | 99.66 |
| 7.0~4.70 | 1.04 | 98.92 |
| 4.70~3.30 | 1.69 | 97.88 |
| 3.30~2.10 | 4.68 | 96.19 |
| 2.10~1.10 | 21.18 | 91.51 |
| 1.10~0.65 | 38.24 | 70.33 |
| 0.65~0.43 | 20.40 | 32.09 |
| < 0.43 | 11.69 | 11.69 |

**Table 2.** Metal and gaseous concentration of MMA-SS welding fumes

| | | Low dose | High dose |
|---|---|---|---|
| Metal ($mg/m^3$) | Fe | 1.32 ± 0.23 | 2.19 ± 0.36 |
| | Cr | 1.00 ± 0.16 | 1.80 ± 0.31 |
| | Mn | 0.65 ± 0.11 | 1.10 ± 0.19 |
| | Ni | 0.12 ± 0.02 | 0.20 ± 0.03 |
| Gas (ppm) | $O_3$ | 0.05 | 0.1 |
| | $NO_2$ | 0.3 | 0.4 |
| | Nitrous fumes | 4 | 8 |

Data is present mean ± S.D.

diameters equal to or less than 2.1 μm. The fume particulates were comprised mainly of Fe, Mn, Cr, and Ni. The concentrations of metal and gaseous fractions of the welding fumes are shown in Table 2.

***Body weight changes and general symptom observations.*** No significant body weight changes were observed throughout the 30 days of exposure or the 30-day recovery period. However, the tendency of decrements in body weight changes was noticed (Fig. 1). Abnormal respiratory sounds such as rale and increased respiration rates were observed in some rats exposed to high-dose welding fumes, but they soon disappeared after the recovery period. No distinct abnormal behavior related to the welding fume exposure was observed.

***Cellular parameters of lung inflammation.*** Table 3 shows the total cell number and differential counts of BAL cells recovered from the lungs of rats at each time point. The numbers of AM, PMN, and lymphocytes were elevated significantly ($p < 0.01$) during the 30 day exposure period in a dose-dependent manner when compared to the control. Fume particle-ingested macrophages and dose-dependent neutrophil increases

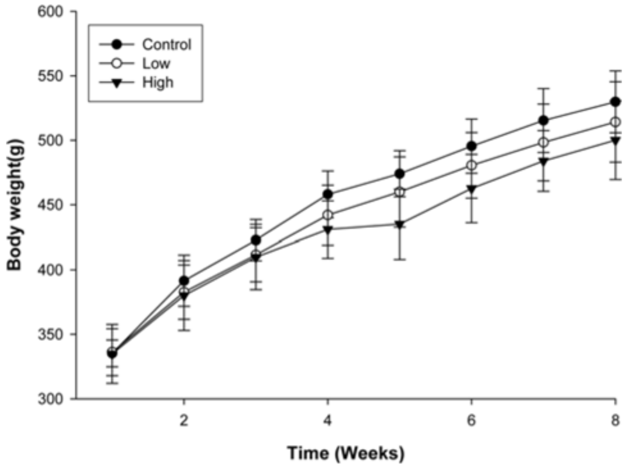

**Fig. 1.** Body weight changes in rats during 30 days of exposure and a 30-day recovery period.

were noted in cytospin images (Fig. 2B, 2C). After the 30-day recovery period, all cell types were decreased significantly ($p < 0.01$) when compared to the exposed group. This observation indicated that the lungs of rats had recovered from pulmonary impairment induced by welding fume inhalation. However, statistically significant increases in the total cell, AM, PMN were found even after the 30-day recovery period and fume particle-laden macrophages were still observed in BAL cells (Fig. 2D).

***Biochemical and cytokine parameters.*** The exposed groups had significant elevations ($p < 0.01$) in the contents of β-NAG and LDH in the BALF when compared to the control group (Fig. 3A, 3B). Particularly, dramatic elevations in LDH activity were observed in a dose-dependent fashion among the 30-day exposure group. Slight, but significant elevations in the β-NAG values were shown following the 30-day welding fume

**Table 3.** BAL cell distribution during 30 days of exposure and a 30-day recovery period

| | | Total cell number (× $10^6$) | | | |
|---|---|---|---|---|---|
| Exposure | Dose | Total | Macrophage | PMN | Lymphocyte |
| 30 days exposure | | | | | |
| | Control | 1.64 ± 0.29 | 1.53 ± 0.27 | 0.04 ± 0.01 | 0.03 ± 0.01 |
| | Low | 8.86 ± 0.47** | 8.09 ± 0.44** | 0.47 ± 0.02** | 0.25 ± 0.01** |
| | High | 17.49 ± 0.71**,a | 14.15 ± 0.66**,a | 2.42 ± 0.02**,a | 0.85 ± 0.03**,a |
| 30 days recovery | | | | | |
| | Control | 1.97 ± 0.19 | 1.89 ± 0.18 | 0.03 ± 0.00 | 0.02 ± 0.00 |
| | Low | 3.87 ± 0.44**,b | 3.53 ± 0.42**,b | 0.08 ± 0.01*,b | 0.05 ± 0.00b |
| | High | 4.43 ± 1.07**,b | 4.00 ± 0.99**,b | 0.12 ± 0.01**,b | 0.08 ± 0.01**,b |

Note. Values are means ± S.D.
*$p < 0.05$ compared with control group. **$p < 0.01$ compared with control group.
[a]$p < 0.01$ low dose vs. high dose. [b]$p < 0.01$ 30 days vs. 60 days.

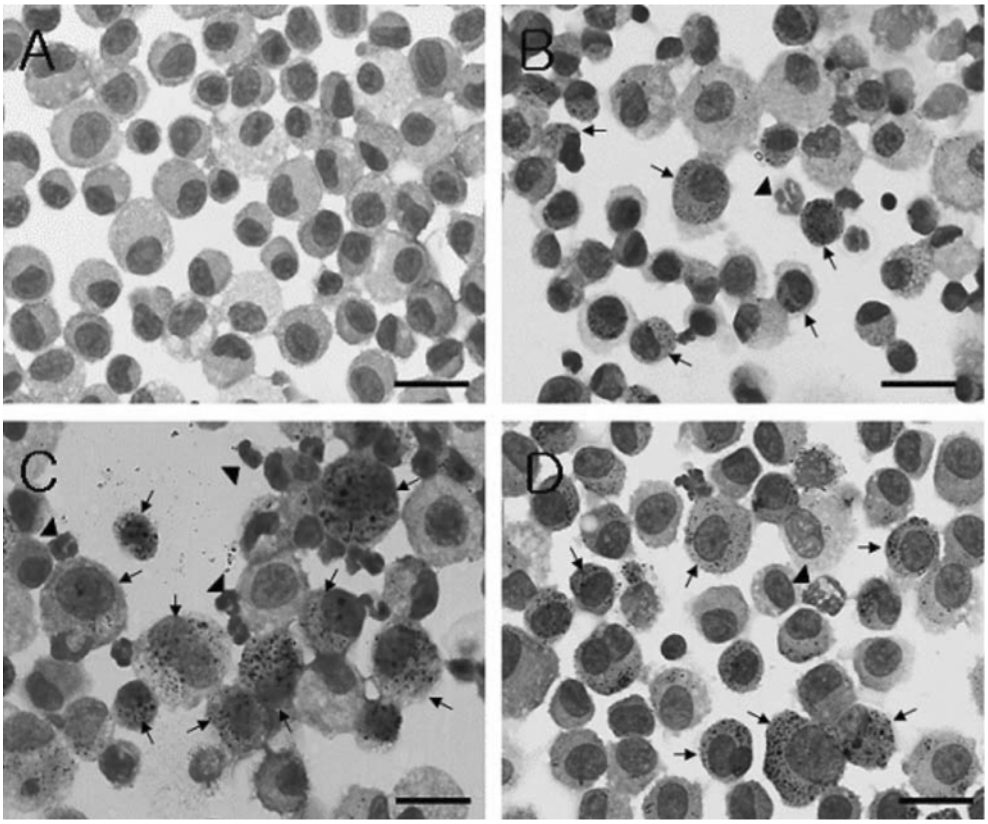

**Fig. 2.** Cytospin images of alveolar cells recovered by BAL from animals. (A) Control, (B) After 30 days low-dose exposure, (C) After 30 days high-dose exposure, and (D) After 30 days recovery of high-dose exposure. Arrows indicate fume particles ingested by alveolar macrophages and arrowheads indicate neutrophils. Bar is 20 μm.

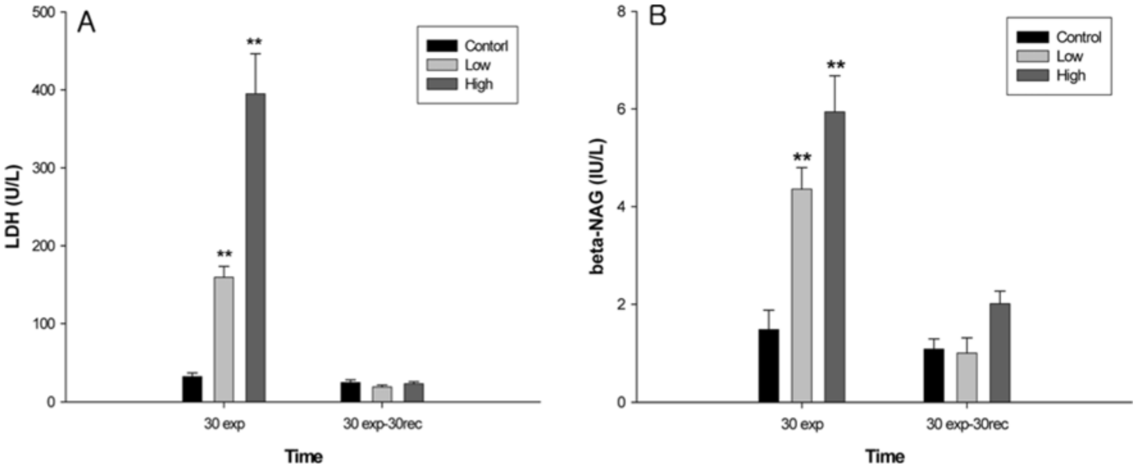

**Fig. 3.** Effect of welding fume exposure and recovery on LDH activity and β-NAG in the BALF.

exposure period. Both β-NAG content and LDH activity had returned to control level values in the recovered groups. No statistically significant changes in the content of albumin or ALP were observed among the groups at any time point (data not shown). The levels of TNF-α and IL-1β in the BALF did not show any statistically significant changes when compared to controls after exposure or recovery periods (data not shown).

***Organ weights, gross observation, and histopathology.*** The left lung weights in high dose exposed rats were significantly increased ($p < 0.01$) versus con-

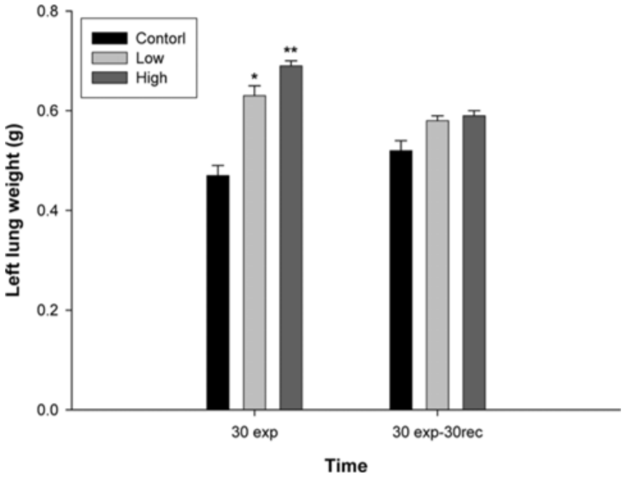

**Fig. 4.** Left lung weight changes during 30 days of exposure and a 30-day recovery period.

trols (Fig. 4), whereas recovered rats did not show any significant body weight changes. The other organ weights, including heart, liver, spleen, kidneys, brain, and testes did not show any significant changes at any time point (data not shown).

In the necropsy, black spots were diffused on the pleural surfaces of the rats exposed to welding fumes and were even more evident on the lungs of 30 day recovered rats. After 30 days of welding fume exposure, numerous fume particle-ingested macrophages and foamy histiocytes were observed in the terminal bronchioles and the gas exchange regions, such as alveolar ducts, alveolar sac, and alveoli. Clusters of the fume particles were present within alveolar macrophages. Overall, cellular debris, mucus granules, and fume particles from burst macrophages filled the alveolar spaces (Fig. 5B).

The number of fume particle-laden macrophages in the alveolar spaces was decreased following 30 days of recovery (Fig. 5C). Cellular debris and mucus granules were rarely found in the alveolar spaces. Most of the fume particles, which were previously retain in the

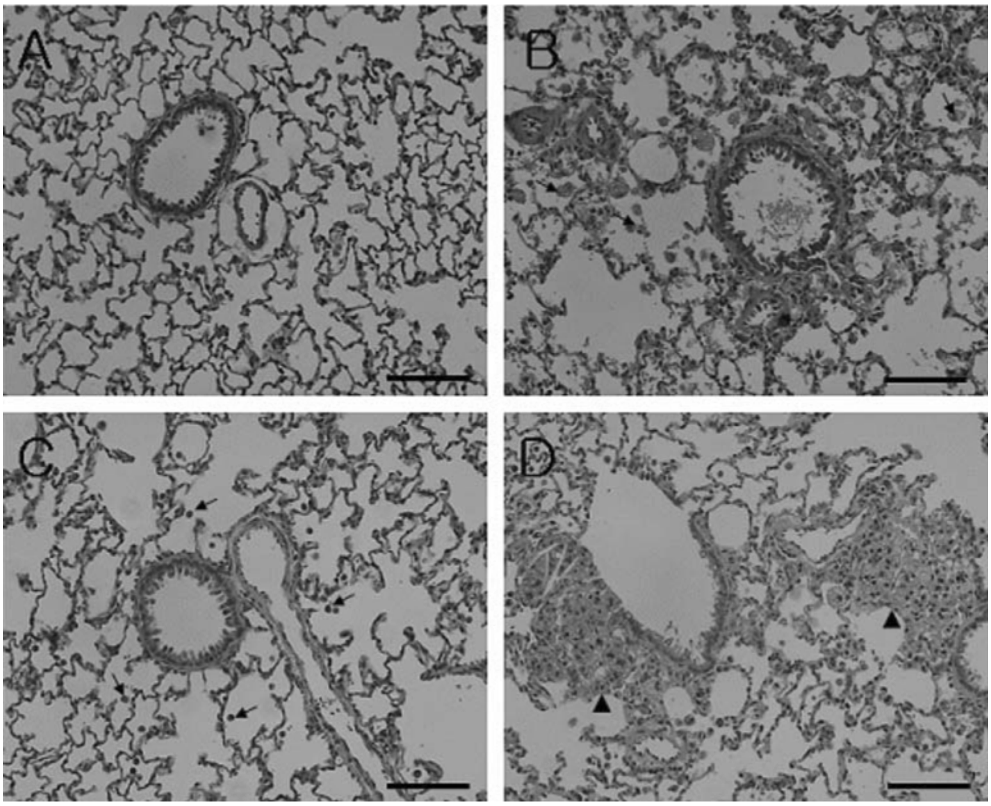

**Fig. 5.** Histopathology of rat lungs exposed to MMA-SS welding fumes for 30-day and a 30-day recovery. (A) Control, (B) after 30 days of exposure, (C) after 30 days of recovery, (D) after 30 days of recovery. Arrows indicate fume particles ingested by alveolar macrophages and arrowheads indicate accumulation of foamy or pigmented macrophages. Bar is 200 μm. (B) Fume particle-ingested macrophages, foamy histiocytes, Cellular debris and mucus granules were observed in the terminal bronchioles and the gas exchange regions after 30 days exposure. (C) Fume particle-laden macrophages, cellular debris and mucus granules in the alveolar spaces were rarely found following 30 days of recovery. (D) Foamy histiocytes and pigmented macrophages had aggregated in nodular-like shapes around the perivascular and peribronchiol regions.

lungs, had been cleared away by the alveolar macrophages after recovery. Foamy histiocytes and pigmented macrophages had aggregated in nodular-like shapes around the perivascular and peribronchiol regions (Fig. 5D). Other significant histopathological abnormal differences were not detected in the exposed and recovered groups. There was no observed histopathological damage to the nasal pathways or other organs.

## DISCUSSION

To investigate the effect of lung inflammatory response and injury, animals were exposed to 51.4 (low dose) or 84.6 (hgih dose) mg/m$^3$ of the MMA-SS welding fumes 2 h per day for 30 days. The rats were then allowed to recover for 30 days to assess the lung repair process. It seems that exposure doses in the current study was too low to evaluate the progression of fibrosis when compared to a previous study (Yu *et al.*, 2001), however they were sufficient to cause significant pulmonary damage and inflammation.

Whole body inhalation exposure system of MMA-SS welding fumes generated by an automatic robotic welder had been developed and used previously (Yu *et al.*, 2007). The inhalation system simulated the actual SS welding processes, so the exposure conditions in this study are similar to the actual welder's workplace. Particle size and components of the MMA-SS welding fumes were determined to be comparable to other studies (Yu *et al.*, 2001, 2007). The mass median aerodynamic diameter size of the welding fumes measured by Anderson sampler was approximately 0.87 μm, and over 90% were less than 2.1 μm. The aerodynamic diameters of welding fume particles in the atmosphere of the welder's breathing zone have been determined to be 0.5~2 μm (Villaume *et al.*, 1979; Voitkevich, 1995). Therefore, it was suggested that the size of the fume particles exposed to the animals in this study are very similar to what is present in the welder's breathing zone.

MMA-SS fumes were comprised mainly of metal constituents including Fe, Cr, Mn, Ni, and gaseous fractions of $O_3$, $NO_2$, and nitrous fumes. Cr and Ni contained in welding fumes has been known to be carcinogenic and shown to be toxic and mutagenic to mammalian cells (Hedenstedt *et al.*, 1977; Maxild *et al.*, 1978). In previous studies, it was reported that both soluble and insoluble fractions of MMA-SS fumes induced oxidative damage to the lungs due to free radical production (Antoninin *et al.*, 1999). Acute lung damage was manifested by increased macrophage and neutrophil numbers as well as pro-inflammatory cytokines (Taylor *et al.*, 2003). Accumulation of Mn in the lungs and brain has been observed following exposure to MMA-SS welding fumes, which indicates a risk of developing manganism in welders (Yu *et al.*, 2003a). The retention and deposition of alloyed metals from SS fumes in the lungs were very high, and were cleared with half-lives of 50, 40, 40, and 30 days for $Fe_{ex}$, Cr, Mn, and Ni, respectively (Kalliomaki *et al.*, 1983). From the previous data, it seems that 30 days for recovery is insufficient to fully clear fume particles retained in the lung.

To evaluate the toxicity of welding fumes, several markers of pulmonary injury and inflammation were measured in the BALF. Increased the level of each indicator was considered to be an index of pulmonary toxicity. MMA-SS fumes inhalation led to a significant increase in cellularity, mainly due to increases in AM, PMN. This indicates that AM and neutrophils are responsible for the inflammatory response towards MMA-SS fumes. The total number of cells was shown following the 30 days recovery, but a significant elevation in AM and PMN was seen at this time point when compared to controls. Fume particles containing AM still existed in the lungs even after 30 days of recovery. The persistence of fume particles in the lungs is considered to be an indicator of continuous particle toxicity. Therefore, it was demonstrated that fume particles remaining in the lungs of rats were not fully cleared and the recovery process from pulmonary inflammation meditated by AM, PMN still continued.

MMA-SS fumes caused a significant increase in the contents of LDH and β-NAG. It represents that the rate of cellular damage and death was increased. LDH and β-NAG activity had returned to control levels after the 30-day recovery period. This might suggest that cellular damage caused by welding fumes had recovered. The increase and decrease in the pulmonary damage markers assayed in the current study were consistent with the changes seen in macrophages, signifying the role of macrophages in mediating the inflammatory responses.

To evaluate the inflammatory cytokines, two cytokines, TNF-α and IL-1β, were measured. None significant changes in TNF-α or IL-1β were observed following 30 days of exposure. This might be due to the role of TNF-α and IL-1β in initiating cytokine cascades, which induce inflammation (Le and Vilck, 1987). On the other hand, other inflammatory agnets besides TNF-α and IL-1β could be involved in persistent pulmonary inflammation. Previously, no difference was observed in TNF-α or IL-1β levels between the fume exposed and control groups (Yu *et al.*, 2003b; Antonini *et al.*, 2007). Also, the marker levels subsided 35 days post instillation after peaking at 7-14 days (Antonini *et al.*, 1996, 1997).

The lung weights was increased following MMA-SS exposure, which equal to the increase in cellularity. An important histopathological finding in this study was the presence of numerous AM phagocytized fume particles and foamy histiocytes throughout the alveolar spaces. Alveolar macrophages are free cells migrating over the surface of the alveolus when stimulated by inhaled dust. Stimulus continues to increase the number of macrophages, which secrete chemoattractants for neutrophils, which accumulated in the alveolar lumen and interstitium. Therefore, the mobilization and recruitment of macrophages to alveolar regions is a significant sign of inflammation. The activated phagocytes, producing reactive oxygen and nitrogen species, could be responsible for oxidative tissue damage after MMA-SS treatment (Taylor *et al.*, 2003). Foamy histiocytes containing lipid droplets within their cytoplasm and brown or black-brown pigmented AM accumulated within peribronchiolar and perivascular regions, indicating the continual phagocytosis of fume particulates. The mucus granules in the alveolar spaces may be correlated with an increase in goblet cells. Other repeated inhalation studies in rats showed histopathological signs of respiratory irritation, such as mucus production in the alveoli and hyperplasia of mucus cells in the bronchial epithelium, which were observed in rats exposed to MMA-SS fumes (Uemitsu *et al.*,1984; Yu *et al.*, 2006).

It was reported that continual exposure to MMA-SS welding fumes induced an early stage of fibrosis, such as peribronchial and perivascular fibrosis, after 30 days (Yu *et al.*, 2001, 2003). Lack of fibrotic changes in the present study might be related to exposure to lower concentrations of fumes than previous studies; therefore, it could be estimated that the concentration and exposure duration in this study was not sufficient to induce the fibrosis in rat lungs (Yu *et al.*, 2001, 2003). In conclusion, the current experimental data clearly demonstrated that welding fume exposure, by inhalation for 30 days, induced damage and inflammation in the lungs and recovery from pulmonary injury was observed 30 days after exposure was stopped.

## ACKNOWLEDGEMENTS

This study was supported by the Ministry of Science and Technology for the General Project grant of the establishment of the International Inhalation Toxicology Evaluation Technology of Korea Institute of Toxicology.
